# Terahertz characterization of two-dimensional low-conductive layers enabled by metal gratings

**DOI:** 10.1038/s41598-021-82560-2

**Published:** 2021-02-02

**Authors:** Prashanth Gopalan, Yunshan Wang, Berardi Sensale-Rodriguez

**Affiliations:** 1 Department of Electrical and Computer Engineering, The University of Utah, Salt Lake City, UT 84112 USA; 2 Department of Chemical Engineering, The University of Utah, Salt Lake City, UT 84112 USA

**Keywords:** Electrical and electronic engineering, Metamaterials

## Abstract

While terahertz spectroscopy can provide valuable information regarding the charge transport properties in semiconductors, its application for the characterization of low-conductive two-dimensional layers, i.e., σ_s_ <  < 1 mS, remains elusive. This is primarily due to the low sensitivity of direct transmission measurements to such small sheet conductivity levels. In this work, we discuss harnessing the extraordinary optical transmission through gratings consisting of metallic stripes to characterize such low-conductive two-dimensional layers. We analyze the geometric tradeoffs in these structures and provide physical insights, ultimately leading to general design guidelines for experiments enabling non-contact, non-destructive, highly sensitive characterization of such layers.

## Introduction

Terahertz spectroscopy has emerged as a contactless, non-destructive technique for characterizing semiconductor materials^1–5^. With the advent of 2D materials, such as graphene and transition metal dichalcogenides (TMDCs), as well as recent progress on two-dimensional sheet charges in semiconductor heterostructures, terahertz spectroscopy of two-dimensional layers has become a topic of particular interest, e.g., Refs.^3,6–10^. In this regard, it is worth noting that at terahertz frequencies, following a Debye model, the charge carrier conductivity is effectively determined over characteristic length scales on the order of nanometers^11,12^. Therefore, the sheet conductivity extracted from terahertz measurements would be a spatially averaged nanoscale conductivity and is minimally affected by microscale scattering phenomena that would play a role in direct current (DC) transport measurements wherein the carrier transport is typically probed over length scales that are three orders of magnitude larger (i.e., micrometers). These could include extended effects such as those arising from point defects or dislocations introduced in epitaxial layers during growth, or grain boundaries usually present in two-dimensional films^12–14^. As a result, charge transport characterization using terahertz spectroscopy can provide a more relevant estimate of such conductive layers' intrinsic carrier transport properties. Additionally, the terahertz-extracted sheet conductivity is more relevant for use in optoelectronic or nano-electronic devices, where the relevant dimensions for charge transport, for instance, dictated by the gate-length in field-effect transistors, are also on the order of nanometers. Furthermore, measuring the frequency dependence of conductivity can also provide valuable information on the physical nature of charge transport as well as dominating scattering mechanisms, e.g., Refs.^15,16^.

**Figure 1:**
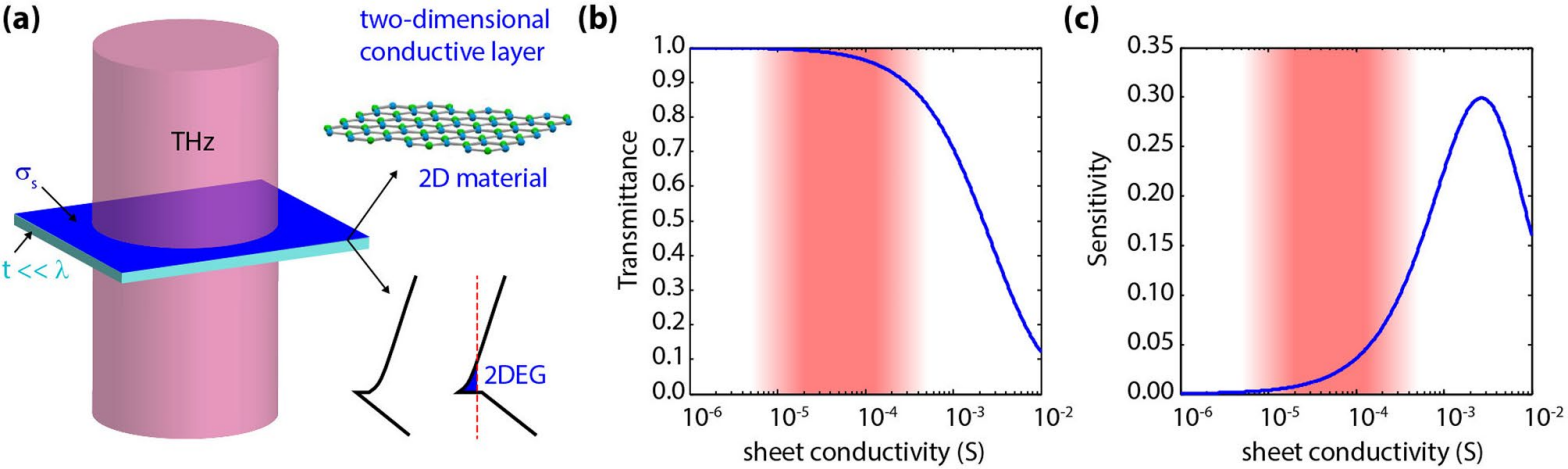
Terahertz transmission through a two-dimensional conductive layer (image created in Adobe Illustrator v25.0.1, https://www.adobe.com/products/illustrator.html). (**a**) Illustration of the analyzed geometry, which consists of a conductive layer with sheet conductivity σ_s_ on top of an optically thin substrate (t <  < λ). (**b**) Calculated transmittance versus sheet conductivity. (**c**) Calculated transmission sensitivity versus sheet conductivity. Sensitivity peaks in the mS range, thus characterizing low-conductive layers (µS range) becomes challenging.

However, the extraction of the terahertz conductivity of such conductive layers from direct transmission measurements is fundamentally limited to a narrow range of conductivity values. This is ultimately dictated by the sensitivity of transmission itself to conductivity. For illustrative purposes, let us consider a two-dimensional conductive layer suspended in free space (or on a very thin substrate). The terahertz beam is normally incident on this layer, as illustrated in Fig. 1a. The layer's sheet conductivity (*σ*_*s*_) is assumed to possess no frequency dispersion in the analyzed frequency range. The transmittance, defined as the ratio between the transmitted powers in the absence (*T*_*0*_) and presence (*T*) of the layer under test, is then given by^17^:

1 $$\frac{T}{{T_{0} }} = \frac{4}{{\left( {2 + Z_{0} \sigma_{s} } \right)^{2} .}}$$

where Z_0_ ~ 377 Ω is the characteristic impedance of free space. Depicted in Fig. 1b is a plot of transmittance versus sheet conductivity (on a semi-log scale). It is observed that at the extrema, i.e., at low conductivity levels (µS range) and high conductivity levels (S range), the transmittance approaches unity and zero, respectively; therefore, measurement and meaningful interpretation of such transmittance levels become quite challenging. By taking the derivative of Eq. (1), with respect to the logarithm of conductivity, one can estimate the associated transmission sensitivity to the sheet conductivity:

2 $$\frac{{d\left( {T/T_{0} } \right)}}{{dLn\left( {\sigma_{s} } \right)}} = { }\frac{{ - 8Z_{0} \sigma_{s} }}{{\left( {2 + Z_{0} \sigma_{s} } \right)^{3} }}.$$

Figure 1c shows the calculated transmission sensitivity versus sheet conductivity. It is observed that as the conductivity approaches zero, the transmission sensitivity also approaches zero. This situation effectively becomes the case when σ_s_ <  < 1/Z_0_ ~ 2.65 mS. From this perspective, it becomes extremely challenging to characterize conductive layers with sheet conductivity levels on the order of µS, which is often the case in single layer TMDCs such as MoS_2_, WSe_2_, etc.^18^ as well as in conductive layers formed in heterostructures such as the two-dimensional hole gas (2DHG) formed at the interface of thin undoped Gallium Nitride (GaN) quantum wells^19,20^. Therefore, in order to be able to characterize the transport properties of such low-conductive two-dimensional layers through terahertz spectroscopy, alternative approaches to direct transmission or reflection spectroscopy must be employed. In this regard, there have been prior works on utilizing parallel-plate waveguide (PPWG) geometries for enhancing this sensitivity^21,22^. By placing a monolayer graphene in the waveguide, the authors demonstrate the detection of very low carrier concentrations on the order of 10^11^ cm^−2^. It is worth noting that such low concentrations are undetectable via simple transmission measurements. While the waveguides offer enhanced sensitivity by increasing the interaction length, the propagating mode must be chosen such that the E-field is parallel to the sheet charge. In this regard, a structure operating in a simple transmission geometry would be very convenient wherein a normally incident beam has its electric field in the plane of the two-dimensional sheet charge.

Recently, metasurfaces have drawn significant attention for terahertz sensing applications, e.g., Refs.^23,24^. Some highly promising geometries consist of extraordinary optical transmission (EOT) structures, e.g., Ref.^25^. However, in contrast to the most widely considered sensing applications, where typically the real part of permittivity is of interest, in this particular scenario of low-conductive two-dimensional layer characterization, the imaginary part of permittivity becomes more relevant. Conceptually, by considering the metamaterial structure's response from an equivalent circuit perspective, we note that introducing a two-dimensional conductive layer manifests as the addition of an equivalent resistance. This contrasts with the commonly analyzed cases of refractive index sensing, wherein the capacitance is altered.

Furthermore, to ensure that the measurement techniques are non-destructive, often direct lithographic patterning of the samples under test is not feasible. Thus, the metasurface needs to be separated from the conductive layer that is being probed by a dielectric spacer. Such a requirement could compromise the application of several structures that have been previously proposed for sensing like, e.g., nanometer-gap split-ring resonators. These requirements are essential to the characterization of low-conductive layers and should be considered and guide the design of extremely sensitive electromagnetic structures.

Electromagnetic (EM) wave transmission through sub-wavelength slits (or gaps) fabricated in metal films have been extensively studied^26–33^. In order to understand the working principle of enhanced transmission through an array (metallic grating), we refer to the terahertz transmission and large E-field enhancement through a single slit shown in Ref.^28^. The working principle of the single slit can be visualized with a simple capacitor model^34^. The terahertz radiation impinging the metal induces surface currents that flow towards the gap and consequently result in charge accumulation at either side of the gap^35^. As the gap width is decreased, the corresponding E-field enhancement in the gap also increases. Interestingly, the magnitude of the Poynting vector is also strongest near the slit. Observations from our previous works on frequency selective surfaces^36^ indicate that in this region, the Poynting vector has strong components directed nearly parallel to the surface (of the substrate and metal plane). This means that light experiences an increased propagation length when compared to a bare sample (wherein the Poynting vector would be perpendicular to the substrate/metal plane). Therefore, due to these two phenomena reinforcing each other, placing a sheet charge in the vicinity of the slit results in a greater interaction with the E-field and consequently larger absorption of THz radiation.

It is important to note that with just a single slit in a metal film, the overall transmittance is significantly diminished. Therefore, one could employ a periodic array of such slits in order to achieve a higher transmittance. Interestingly, the E-field enhancement is reduced when the distance between the adjacent slits is reduced since the charge collected per gap also reduces. We choose an optimal period and gap size to achieve a larger far-field transmission while producing sufficient E-field enhancement in the slit. Notice that the smallest achievable gap is set by the available lithography technique, typically optical lithography, and is set at 2 µm for practical design considerations, although the effect of slit width is also studied in this work. Through a systematic study of different grating geometries and a thorough analysis of their tradeoffs, we identify several promising geometries and provide general design guidelines for the terahertz characterization of low-conductive layers.

## Methods

Full-wave electromagnetic simulations were performed employing Ansys HFSS^37^. In order to provide a qualitative insight and ease the computational requirements, in most of our examples, the conductive layer was assumed to be surrounded by free space. However, the discussion and conclusions here also apply to the most general cases where a substrate and multiple thin dielectric layers (thickness <  < λ) are present.

For the purpose of simulation, the conductive layer was assumed to be two-dimensional and defined as a '*layered impedance'* boundary condition. We have employed this approach in the past. In those previous works, we have demonstrated an excellent agreement between experimental measurements, full-wave simulations assuming a finite thickness, and theory, e.g., Refs.^38,39^. Additionally, to further simplify the simulations, during the first part of our discussion, the metal layer is assumed to be a perfect electric conductor (PEC). In reality, the finite conductivity of the metal will behave as an additional source of loss and reduce the transmission levels and quality factors of the resonances and alter the sensitivity of the structure (as shown in later sections).

## Results and discussion

**Figure 2:**
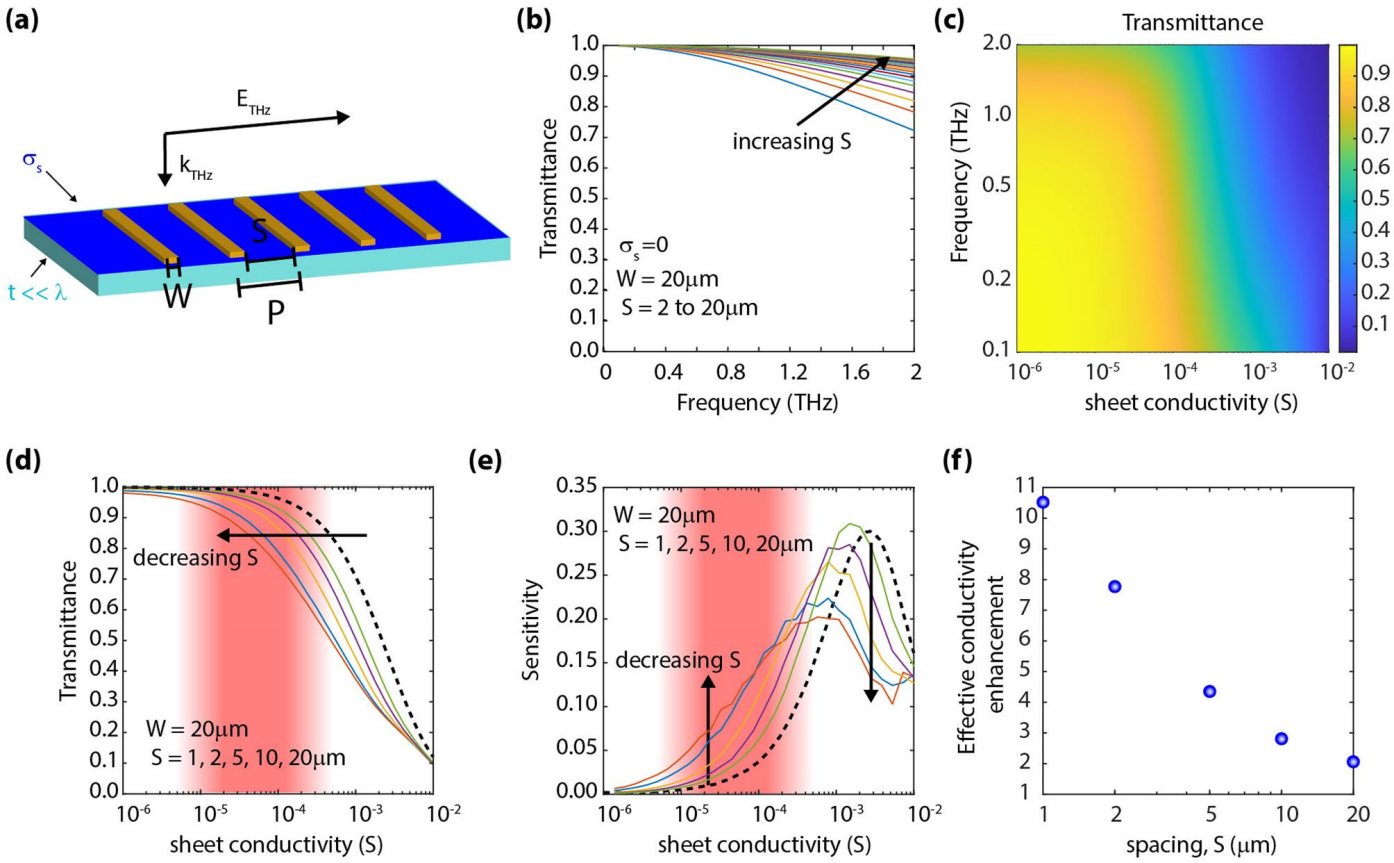
Effect of incorporating a non-resonant grating. (**a**) Illustration of the analyzed grating structure. (image created in Adobe Illustrator v25.0.1, https://www.adobe.com/products/illustrator.html). (**b**) Transmittance spectra, in the absence of a conductive layer (i.e., σ_s_ = 0), when varying S (W = 20 µm). (**c**) Colormap of transmittance versus frequency and sheet conductivity. (**d**) Passband (i.e., *f* < 0.2 THz) transmittance versus sheet conductivity. The dashed black curve corresponds to the case depicted in Fig. 1b; continuous colored curves correspond to S = 1, 2, 5, 10, and 20 µm. Decreasing S pushes the characteristic curves towards lower conductivity levels. (**e**) Transmission sensitivity versus sheet conductivity. The dashed black curve corresponds to the case depicted in Fig. 1c; continuous colored curves correspond to S = 1, 2, 5, 10, and 20 µm. Decreasing S increases the sensitivity at low conductivity levels as well as shifts the position of the maxima. (**f**) Effective conductivity enhancement versus spacing, S. The smaller the spacing, the larger the enhancement.

We analyzed the response of a grating structure, as depicted in Fig. 2a. For this analysis, we varied the width (W) of the parallel metallic stripes and the spacing (S) between them. We also explored the effect of a finite metal conductivity on the structure's response. Such structures have been previously analyzed in the context of dielectric sensing, e.g., Refs.^40–42^, as well as more recently for terahertz modulation by graphene^43^. The response of this structure has two different regimes, a resonant one when the period (P) is P >  > λ, and a non-resonant one, when P <  < λ.

### Non-resonant regime

When P <  < λ, the transmission response of the grating follows that of a low pass filter. Therefore, a broadband region with high transmittance (passband) with a roll-off at higher frequencies is observed, as illustrated in Fig. 2b. It is worth noticing in Fig. 2b that as the spacing (S) is increased, while maintaining the stripe width (W) constant, the roll-off shifts towards higher frequencies. This can be understood from the roll-off frequency being inversely proportional to the gap-capacitance, which decreases when increasing the spacing (S). The addition of the conductive layer further alters the transmission levels in the passband. This is seen in Fig. 2c, which depicts the simulated transmittance spectra while varying sheet conductivity (for S = 2 µm). Figure 2d shows the transmittance versus sheet conductivity at the passband when the spacing (S) is varied while keeping the width (W) constant. It is observed that as the spacing, S, is decreased, the curves shift towards lower conductivity levels. This effect is further elaborated in Fig. 2e, which depicts the transmission sensitivity for different values of S. Physically, this can be understood from the perspective of an effective conductivity enhancement, which directly follows the field enhancement, as discussed by Yan et al*.*^38^. Shown in Fig. 2f is the extracted conductivity enhancement, defined as the ratio between the actual layer conductivity and the conductivity level that would provide an equivalent drop in transmission in the case where no grating is present. We observe that the smaller the spacing, the larger the enhancement. These observations are in good agreement with the previous studies reported by Lee et al*.* on graphene modulators enhanced through EOT gratings^43^. However, to push this region of high sensitivity to the region of lower conductivity values (red shaded region in Figs. 1b,c and 2d,e), the dimension of the spacing needs to be reduced well below 1 µm. Achieving sub-micron dimensions is challenging from the perspective of fabrication and therefore limits the application of this design.

**Figure 3:**
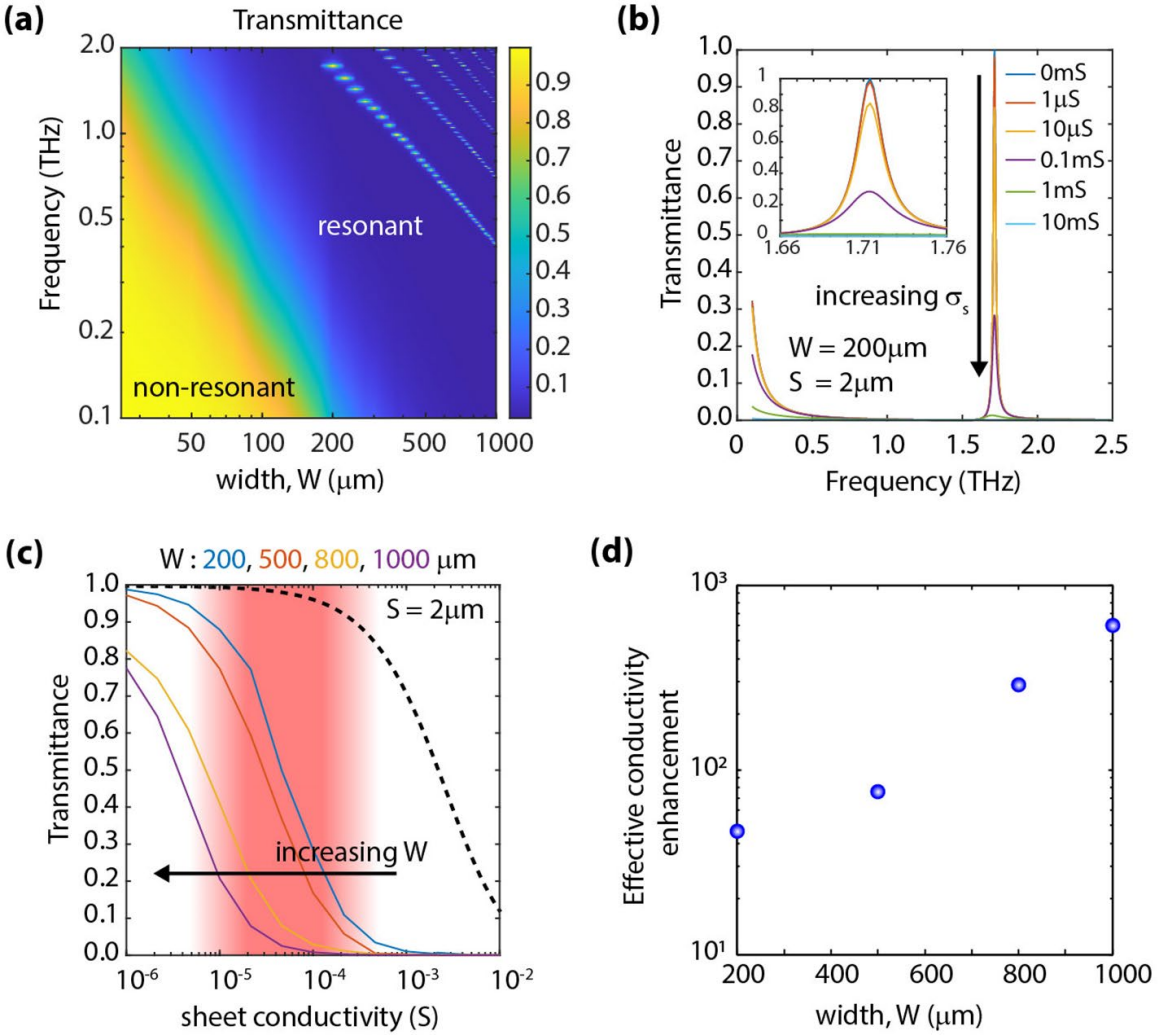
Effect of incorporating a resonant grating. (**a**) Colormap of transmittance versus frequency and stripe width, W; S was fixed to S = 2 µm in the absence of a conductive layer (σ_s_ = 0). (**b**) Transmittance spectra, when employing a grating with W = 200 µm and S = 2 µm for various sheet conductivity levels, σ_s_. (**c**) Transmittance (at resonance) versus sheet conductivity. The dashed black curve corresponds to the case depicted in Fig. 1b; continuous colored curves correspond to W = 200, 500, 800, and 1000 µm, respectively. Increasing W shifts the characteristic curves towards lower conductivity levels. (**d**) Effective conductivity enhancement versus stripe width, W.

It is worth noticing that in contrast to the behavior observed in Fig. 2b, if the metal stripe width (W) is increased while keeping the spacing (S) constant, as will be later shown in Fig. 3a, one would observe a red shift in the roll-off frequency (i.e., a shift towards lower frequencies). After considering these trends, we can conclude that in addition to the insufficient conductivity enhancement without sub-micron spacing, the frequency red-shift of the passband region causes the region of high E-field transmission to fall out of the terahertz range (i.e., 0.1–2 THz), which would preclude us from employing the non-resonant region of operation as an effective way to characterize low-conductive layers.

### Resonant regime

In general, increasing the filling factor, defined as *f* = W/P, is critical to enhancing the effective conductivity. We performed simulations keeping S constant (S = 2 µm) and varying W for analyzing this effect. In this case, we observe that as W is increased, the transmission response transitions from a non-resonant regime to a resonant response when P becomes comparable to the incident terahertz wavelength, as shown in Fig. 3a. This behavior is well known from grating theory and was observed in our and other previously reported works^44^. The colormap plot shown in Fig. 3a depicts transmission levels at different frequencies versus stripe width (W). We can clearly observe that for small values of W, thus P <  < λ, the structure responds as a low-pass filter as discussed in the previous section. The roll-off of this low-pass filter response shifts towards lower frequencies as W is increased. Upon increasing W, as W becomes on the order of λ, we start to observe a series of transmission peaks (resonances). To understand the effect of incorporating a conductive layer on the transmission spectra, additional simulations were performed by varying W and *σ*. We observed that for all values of W, the transmittance amplitude at the resonance is altered when the sheet conductivity (*σ*_*s*_) is varied, while the position of the resonance does not significantly shift. However, the position of this resonance does strongly shift with W. Depicted in Fig. 3b is the transmission spectra for a grating with W = 200 µm, the transmission is very sensitive to the conductivity of a layer placed in its proximity. Figure 3c shows the extracted family of curves representing transmittance levels (at the resonance) versus layer sheet conductivity for different values of W. It is observed that as the filling factor is increased (by increasing W), the transmittance characteristics shift towards lower conductivity levels. In this case, the curves effectively shift to the region of interest (depicted by the red shaded region). The most notable aspect of this design is that when W = 1000 µm and S = 2 µm, an exceptional, ~ 600 × enhancement is observed for an underlying sheet conductivity of ~ 50 µS as depicted in Fig. 3d.

### Effect of spacing between the grating and conductive layer

**Figure 4:**
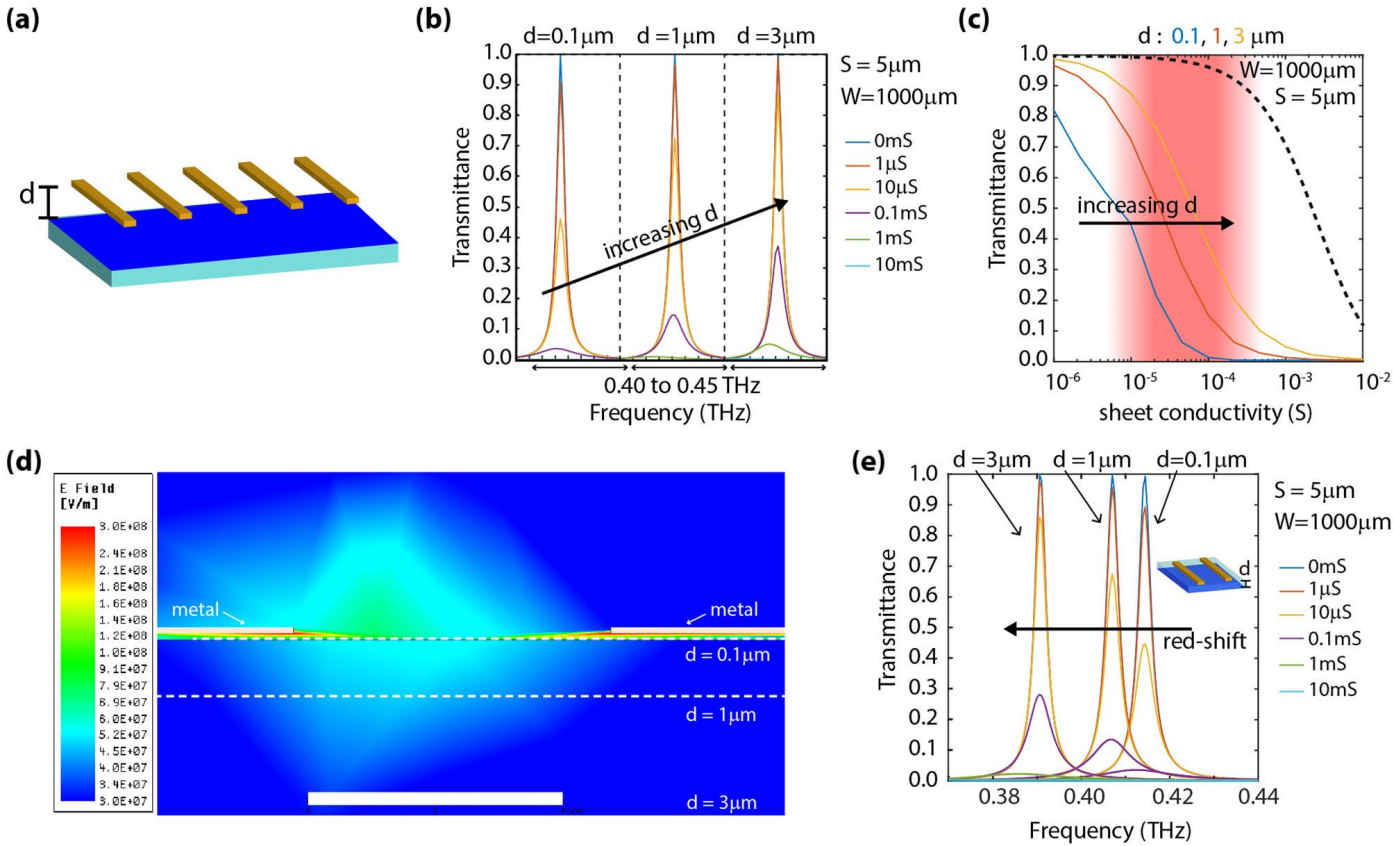
Effect of spacing between the grating and conductive layer. (**a**) Illustration of the analyzed geometry. We vary the spacing, d, between the grating and structure under test in our simulations. (image created in Adobe Illustrator v25.0.1, https://www.adobe.com/products/illustrator.html). (**b**) Transmittance spectra (when varying sheet conductivity) for d = 0.1, 1, and 3 µm. (**c**) Transmittance (at resonance, i.e., ~ 0.42 THz) versus sheet conductivity. The dashed black curve corresponds to the case depicted in Fig. 1b, colored continuous curves correspond to d = 0.1, 1, and 3 µm, respectively. Increasing d shifts the characteristic curves towards higher conductivity levels. (**d**) Simulated field distribution (at resonance) in the gap of the structure. The white scale bar represents 4 µm. (**e**) Assuming a polyimide spacer (light blue layer) between the grating and conductive layer, transmittance spectra (when varying sheet conductivity) for d = 0.1, 1, and 3 µm. A redshift is observed in the position of the resonance as d is increased.

In this section, we discuss the effect of altering the spacing (d) between the grating and the conductive layer, as illustrated in Fig. 4a. Depicted in Fig. 4b is the simulated transmittance spectra for d = 0.1, 1, and 3 µm. It is observed that when increasing d, the transmission levels at resonance increase. This is also illustrated in Fig. 4c, which depicts transmittance (at resonance) versus sheet conductivity. The insertion of a few micrometers (µm) thick spacing alters the results by introducing a shift in the most sensitive region towards larger conductivity levels. Again, this could be understood based on a smaller field enhancement when d is increased, as depicted in Fig. 4d. However, this region still remains in the μS range. From this perspective, as well as that of achievable dimensions employing optical lithography, grating structures consisting of large metal filling factors, *f* > 0.99, and moderate gaps, S ~ 2–5 µm, can be a good choice for sensing low conductive layers. It is worth noting that since the response of these gratings is strongly polarization-dependent, the conductivity characterization can be performed only along a particular direction (i.e., along the direction perpendicular to the grating stripes).

While in the previous paragraph we discussed the effect of spacing between the grating and conductive layer assuming free-space as the medium in-between, a practical scenario would have to account for a dielectric spacer between the grating and the conductive layer. To analyze this effect, we performed simulations assuming the spacer to be a polyimide (ε_r_ ~ 3.35) layer. Depicted in Fig. 4e is the simulated transmittance spectra for d = 0.1, 1, and 3 µm. It is observed that the insertion of a few micrometers (µm) thick dielectric alters the results by not just introducing a shift in the most sensitive region towards larger conductivity levels (as previously discussed in Fig. 4b) but also by red shifting the position of the resonance. However, when analyzing the variations in transmittance at the resonance frequency of the structure, the observations and trends remain similar to those for the case represented in Fig. 4b.

### Practical considerations

**Figure 5:**
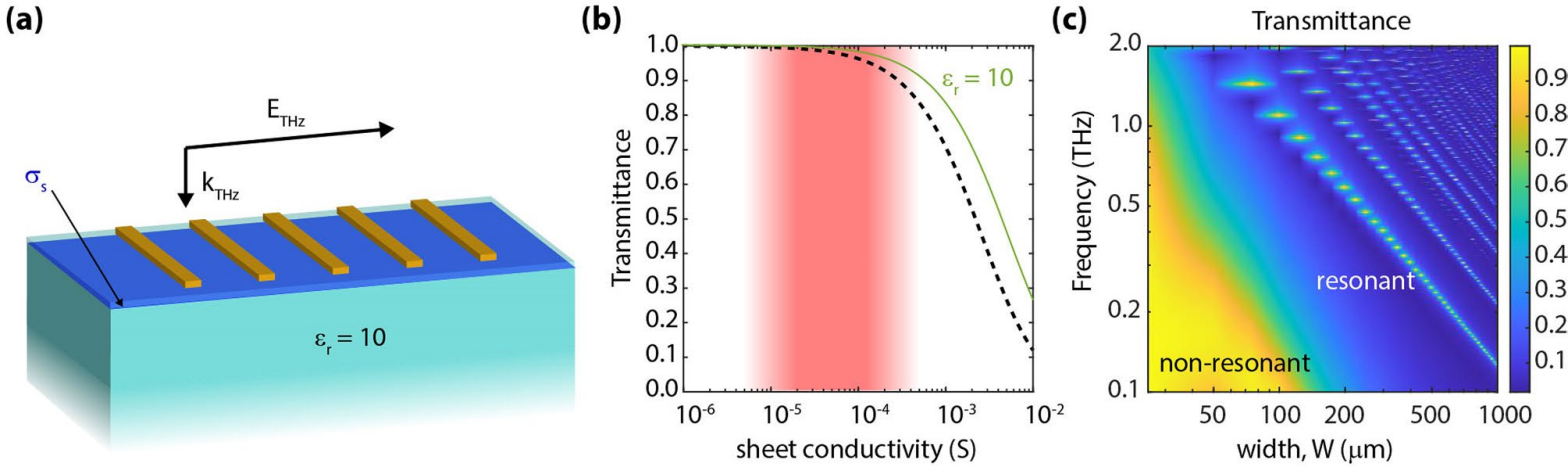
Effect of an optically thick substrate on the layer and grating response. (**a**) Illustration of the analyzed geometry. (image created in Adobe Illustrator v25.0.1, https://www.adobe.com/products/illustrator.html). (**b**) Transmittance versus sheet conductivity for a dispersion-free conductive layer suspended in free-space (dashed black curve) and on top of a semi-infinite substrate with relative permittivity ε_r_ = 10 (continuous green curve). Presence of a substrate shifts the characteristic curve towards higher conductivity levels. (**c**) Colormap of transmittance versus frequency and stripe width, W; S was fixed to S = 2 µm in the absence of a conductive layer, i.e., σ_s_ = 0. Presence of a substrate redshifts the resonances with respect to the situation simulated in Fig. 3a.

To illustrate the application of this grating in a realistic scenario, we performed simulations of a multilayer structure consisting of a two-dimensional conductive layer embedded within a sapphire substrate (ε_r_ ~ 10). The distance between the top surface and the conductive layer was set to 100 nm. An illustration of the analyzed geometry is shown in Fig. 5a.

### Effect of the substrate on the layer and grating response

Figure 5b shows the simulated transmittance (normalized to that of a bare sapphire substrate) versus sheet conductivity of the two-dimensional layer (solid green curve). For reference, the transmittance of a suspended conductive layer is shown in the same plot (dashed black curve). It is observed that the addition of a substrate shifts the curve towards larger conductivity levels, thus effectively reducing the sensitivity of the transmittance to low conductivities (red shaded region). We also performed simulations for a structure consisting of a grating on top of the sapphire substrate. The metal layer was taken as PEC, and no conductive layer was considered. A colormap of transmittance versus width of the metal stripes and frequency is depicted in Fig. 5c. The spacing, S, between adjacent stripes was set to 2 µm. When comparing the results in Fig. 5c from those in Fig. 3a, where no substrate was considered, we observed a redshift of the resonances (as expected from a higher permittivity dielectric environment). It is important to note that the substrate here is taken to be semi-infinite. In comparison, in the previous sections, the conductive layer was assumed to be suspended.

### Effect of metal losses (finite conductivity) on the grating response

**Figure 6:**
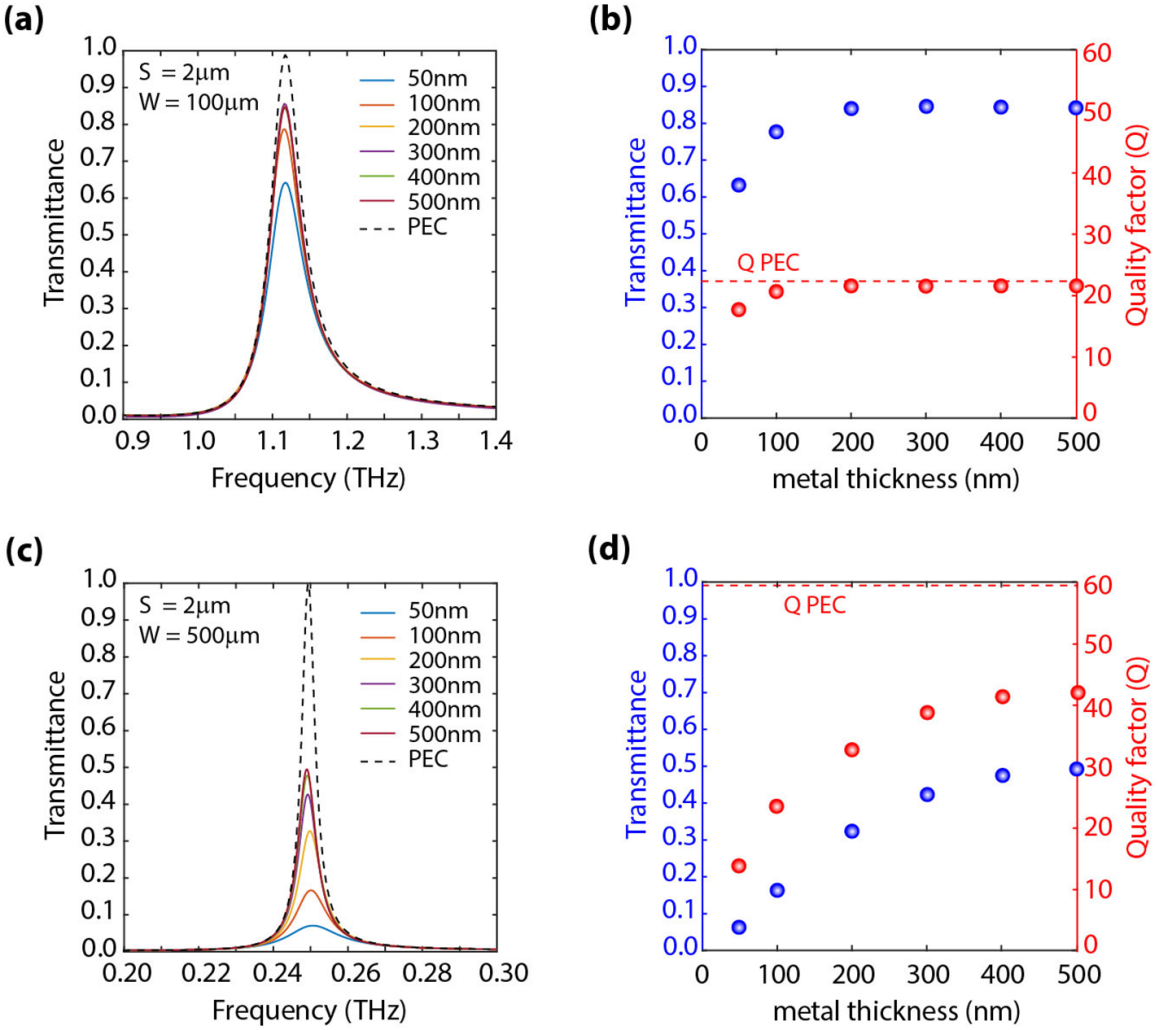
Effect of metal losses (finite conductivity) on the grating response. (**a**,**c**) Simulated transmittance spectra for different metal thicknesses (gold) for a grating with (**a**) W = 100 µm and (**c**) 500 µm. In both cases, S was set to 2 µm. (**b**,**d**) Transmittance spectra at resonance, i.e., ~ 1.1 THz and ~ 0.25 THz, when varying metal thickness for a grating with (b) W = 100 µm and (**d**) 500 µm. When comparing (**b**,**d**) is observed that the larger the stripe width, W, the larger the quality factor, Q, of the resonance; however, the larger the effect of metal losses, which leads to a decrease in transmission levels at the resonance as W is increased.

To analyze the effect of metal losses on the grating response, we performed simulations assuming a finite conductivity of *σ*_*m*_ = 4.1 × 10^7^ S/m (corresponding to gold). Shown in Fig. 6a is the simulated transmittance for different gold metal thicknesses (50, 100, 200, 300, 400, and 500 nm), depicted as continuous curves and, for the PEC depicted by the dashed curves. Simulations were performed for a grating with W = 100 µm and S = 2 µm placed on top of a sapphire substrate. Depicted in Fig. 6b is the extracted peak transmittance and quality factor of the resonance versus metal thickness. In general, we observe that the thinner the metal, the lower the peak transmittance, and the lower the quality factor. This can be understood on the basis of a larger loss introduced by the thin metal layer. However, as the metal thickness is increased, the transmittance and quality factors start saturating. This is due to the results becoming independent of the metal thickness as it becomes significantly larger than the skin depth in the metal. A similar analysis was performed for a grating with W = 500 µm and S = 2 µm placed on top of a sapphire substrate. The results are depicted in Fig. 6c,d. Although similar trends are observed to those in Fig. 6a,b, a larger decrease of peak transmittance with respect to that of PEC is observed in this situation. This can be understood from the perspective that larger quality factors in this structure lead to a larger sensitivity to metal losses. Therefore, in order that the transmittance level does not significantly drop from unity, a metal layer thickness of ~ 500 nm is required.

### Analysis of a structure considering these effects

**Figure 7:**
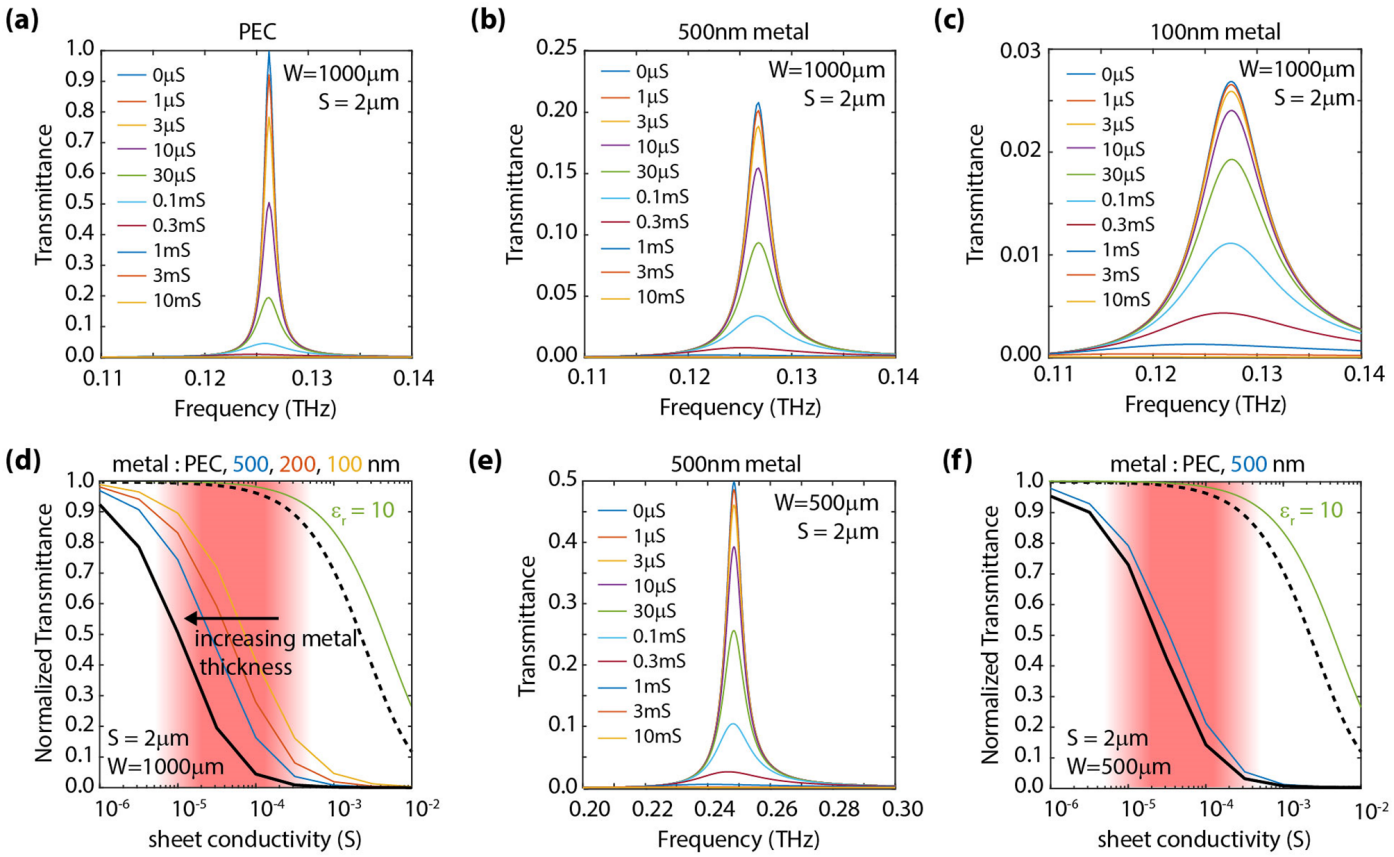
Analysis of sensitivity considering the combined effect of metal losses and the presence of a substrate. (**a–c**) Simulated transmittance spectra when varying sheet conductivity when employing a grating with W = 1000 µm and S = 2 µm. Panels (**a**–**c**) correspond to: (**a**) PEC, (**b**) 500 nm metal, and (**c**) 100 nm metal, respectively. (**d**) Normalized transmittance (at resonance, i.e., ~ 0.13 THz) versus sheet conductivity. The dashed black curve and continuous green curves correspond to the cases depicted in Fig. 5b; the continuous black curve corresponds to PEC while the continuous blue, orange, and yellow curves correspond to gold metal of different thicknesses. Increasing the metal thickness shifts the characteristic curves towards lower conductivity levels. (**e**) Transmittance spectra when varying sheet conductivity when employing a grating with W = 500 µm and S = 2 µm. (**f**) Normalized transmittance (at resonance, i.e., ~ 0.25 THz) versus sheet conductivity.

We performed simulations to analyze the performance of a structure considering these practical effects, i.e., the presence of a substrate and the effect of metal thickness. The results are summarized in Fig. 7. In order to analyze the effect of metal thickness on the structure response, we performed simulations for PEC (Fig. 7a), as well as 500 nm (Fig. 7b) and 100 nm (Fig. 7c) gold. For this purpose, a grating with W = 1000 µm and S = 2 µm was considered. The conductivity was varied from 0 to 10 mS. Peak transmittance levels are observed to significantly drop as a result of the metal losses. However, a large sensitivity is observed in all the cases when normalizing the transmittance levels for the grating in the presence of a conductive layer over those of the same substrate without the conductive layer (as shown in Fig. 7d).

Furthermore, as observed in Fig. 7d, when plotting peak normalized transmittance vs. sheet conductivity, we observe that increasing the metal thickness does not only increase the transmission levels but also shifts the transmittance curves towards lower conductivity levels. Similar trends are observed for a W = 500 µm structure, as depicted in Fig. 7e (transmittance spectra) and Fig. 7f (peak transmittance versus sheet conductivity). Such a structure provides excellent sensitivity over the whole conductivity region of interest (1μS to 1 mS) and adequate peak transmission levels, close to ~ 50%, in the absence of a conductive layer.

## Conclusions

In conclusion, we demonstrate that the light-matter interaction can be considerably increased by integrating metallic gratings to low-conductive two-dimensional layers due to a strong field enhancement. This approach could be utilized just for its field enhancement alone, e.g., broadband subwavelength gratings with small spacing between parallel metallic stripes, or as a combination of field enhancement and a large quality factor in its resonant operation region to obtain a strong sensitivity of the terahertz transmission to the low-conductive layers under test. Additionally, one could potentially extract the dispersion in conductivity in such two-dimensional systems by either utilizing the broadband non-resonant design or the multiple resonances of this structure in the resonant region of operation. Utilizing numerical simulations, we demonstrate that in these structures, it is possible to effectively increase conductivity levels that are originally in the µS range by more than two orders of magnitude. Furthermore, this can be achieved in structures with minimum feature sizes greater than 1 µm, which could be spaced a few micrometers (µm) away from the layer under test, thereby reducing the burden on the lithography and fabrication steps. Such designs enable the non-destructive terahertz characterization of such layer sheet conductivity. Furthermore, the general guidelines discussed here can also find an application on the design of reconfigurable devices, such as modulators, employing low conductive layers such as TMDCs by means of overcoming the modulation depth versus speed tradeoff in these^45^ through effectively boosting the layer conductivity and effective conductivity swing.
